# Aldehyde dehydrogenase 1 expression in primary and metastatic renal cell carcinoma: an immunohistochemistry study

**DOI:** 10.1186/1477-7819-11-298

**Published:** 2013-11-22

**Authors:** Samuel Abourbih, Kanishka Sircar, Simon Tanguay, Wassim Kassouf, Armen Aprikian, Jose Mansure, Fadi Brimo

**Affiliations:** 1 Department of Urology, McGill University Health Center, Montreal, QC, Canada; 2 Department of Pathology, The University of Texas MD Anderson Cancer Center, 1515 Holcombe Blvd, Houston, TX 77030, USA; 3 Department of Pathology, McGill University Health Center, Montreal, QC, Canada

**Keywords:** ALDH1, Renal cell carcinoma, Prognosis, Immunohistochemistry

## Abstract

**Background:**

ALDH1 has been shown to be a cancer stem cell marker, and its expression correlates with prognosis in a number of malignancies. We aimed to evaluate the expression of ALDH1 in a cohort of primary and metastatic RCC specimens, and to correlate expression with pathological outcomes such as tumor stage and grade, and clinical outcomes such as progression free survival.

**Methods:**

Three tissue microarrays were constructed from 244 RCC specimens, taken from 1985 to 2006. Samples were stained using an ALDH1 monoclonal antibody and expression was quantified by degree of staining. Membrane and cytoplasm staining were considered separately. A retrospective chart review enabled correlation with clinical outcomes.

**Results:**

ALDH1 expression did not vary significantly based on tumor stage (*P* = 0.6274) or grade (*P* = 0.1666). ALDH1 showed significantly more membranous expression in clear cell RCC versus other subtypes (*P* < 0.0001), as well as in the primary setting compared to metastases (*P* = 0.0216). In terms of progression free survival, no significant differences were seen based on ALDH1 expression levels. In a subanalysis of clear cell tumors, ALDH1 membranous expression was decreased in tumors of higher stage (*P* = 0.0233).

**Conclusions:**

ALDH1 may be useful in characterizing RCC tumors as clear cell subtype. However, unlike in other malignancies, ALDH1 may not be useful in prognosticating renal cancers. The clinical significance of decreased ALDH1 expression in the high stage and metastatic setting remains to be determined in further investigations.

## Background

Renal cell carcinoma (RCC) is a diverse disease in its histological appearance and biological behavior. While small, organ-confined tumors tend to have excellent cure rates, there is unfortunately no curative treatment for metastatic RCC at present. Because locally advanced RCC is a heterogeneous group in terms of its metastatic potential and overall survival, it is important for clinicians to identify patients who are at high risk of recurrence or metastases so that they can be followed closely and managed more aggressively. Recently, certain molecules have come to the fore because of their roles in tumorigenesis and tumor progression such as the tetraspanins [1], CD133 [2] and the aldehyde dehydrogenase enzymes [3]. These molecules have been the subject of intense scrutiny in the hopes of developing targeted therapies which may slow or arrest the malignant process. In the current study, we have evaluated one of these molecules, aldehyde dehydrogenase 1 (ALDH1) in a large cohort of primary and metastatic RCC specimens.

The aldehyde dehydrogenase family is a large superfamily of proteins, ubiquitously expressed in mammals, which serve a number of important functions. These enzymes are found in several subcellular compartments, including the cytosol, nucleus and endoplasmic reticulum [4]. In addition to converting toxic aldehydes to their corresponding carboxylic acids, ALDH enzymes have ester hydrolysis and nitrate reductase activity. These enzymes also bind endo- and xenobiotics and have structural roles. Defects in various ALDH enzymes lead to diseases such as spina bifida, ethanol-induced cancers, Sjögren-Larsson syndrome and others [5].

The emergence of cancer stem cell theory has led to increased focus on one subtype of ALDH, ALDH1. ALDH1 has been identified as a cancer stem cell marker in a number of different solid tumors including breast, prostate, and bladder [6-9]. In producing retinoic acid, an important regulator of cellular differentiation, ALDH plays a role in cellular differentiation. To lend further proof to this concept, inhibition of ALDH with diethylaminobenzaldehyde has been shown to delay the differentiation of hematopoietic stem cells in culture [10]. In the prostate cancer model, compared with cells weakly expressing ALDH, ALDH highly-expressing cells were significantly enriched in the same antigens expressed by basal cells [6]. This basal layer of cells is the presumed location of prostate stem cells. In addition these high ALDH expressing cells also had abundant anti-apoptotic and detoxifying enzymes such as Bcl-2 and ABCG2, which may help ensure survival of stem cells [6].

While various markers have been studied in renal cancer stem cells [11], the clinical significance of ALDH1 has not been well elucidated. Our aim was to evaluate the immunohistochemical expression of the ALDH1 in primary and metastatic RCCs, especially of the clear cell type, and to assess whether it is associated with local tumor characteristics. We also examined whether this marker could be prognostic by correlating its expression with clinical outcome.

## Methods

### Case characteristics

Three tissue microarrays (TMAs) were constructed. The tissues were obtained from pathologic specimens at the McGill University Health Center, using institutional review board-approved informed consent. Specimens were obtained from nephrectomies performed between 1985 and 2006. The case characteristics are summarized in Table 1.

**Table 1:** Baseline patient characteristics

	**Low stage/High stage TMA**	**Matched primary, metastasis TMA**	**Metastasis TMA**
Age at time of surgery	60.1	49.8	52.9
Percent female	35.4	37.0	n/a
RCC subtype N (%)			
Clear cell	68 (82.9%)	23 (85.2%)	128
Papillary	12 (14.6%)	3 (11.1%)	5
Percent > stage T1	37.8%	n/a	n/a
Percent > grade II	28.0%	44.4%	n/a
Metastasis location	n/a	^a^	
*Lymph node*		7	34
*Bone*		1	25
*Brain*		1	21
*Lung*		3	20
*Adrenal*		4	11
*Other*		3	18

^a^Not all locations were available from chart review.

The first TMA contained RCC specimens from 82 patients, 31 of which had American Joint Committee on Cancer [12] stage T3 or higher (high stage cohort), and 51 with stage T1a disease (low stage cohort). Sixty-three of the specimens were clear cell RCC and 13 were papillary RCC. One specimen containing carcinoid tumor was excluded from analysis. The second TMA contained specimens from 27 patients with metastatic RCC. Each patient in the TMA had a core specimen taken from their primary malignancy, and a matched core specimen from their metastasis. Twenty-three of the specimens were of the clear cell type, while three were papillary and one was unclassified RCC. Sites of metastasis included lymph nodes (n = 7), adrenal glands (n = 4), lung (n = 3), liver (n = 2), bone (n = 1), brain (n = 1) and omentum (n = 1). The third TMA consisted of 135 core specimens of metastatic RCC. Five of the specimens were papillary RCC, and one was chromophobe. The remaining specimens contained clear cell RCC. There were 34 specimens from lymph nodes, 25 specimens from bone, 21 from brain, 20 from lung, 11 from adrenals, 4 from skin, 2 from thyroid, and 12 from miscellaneous other sources.

### Immunohistochemistry

**Figure 1:**
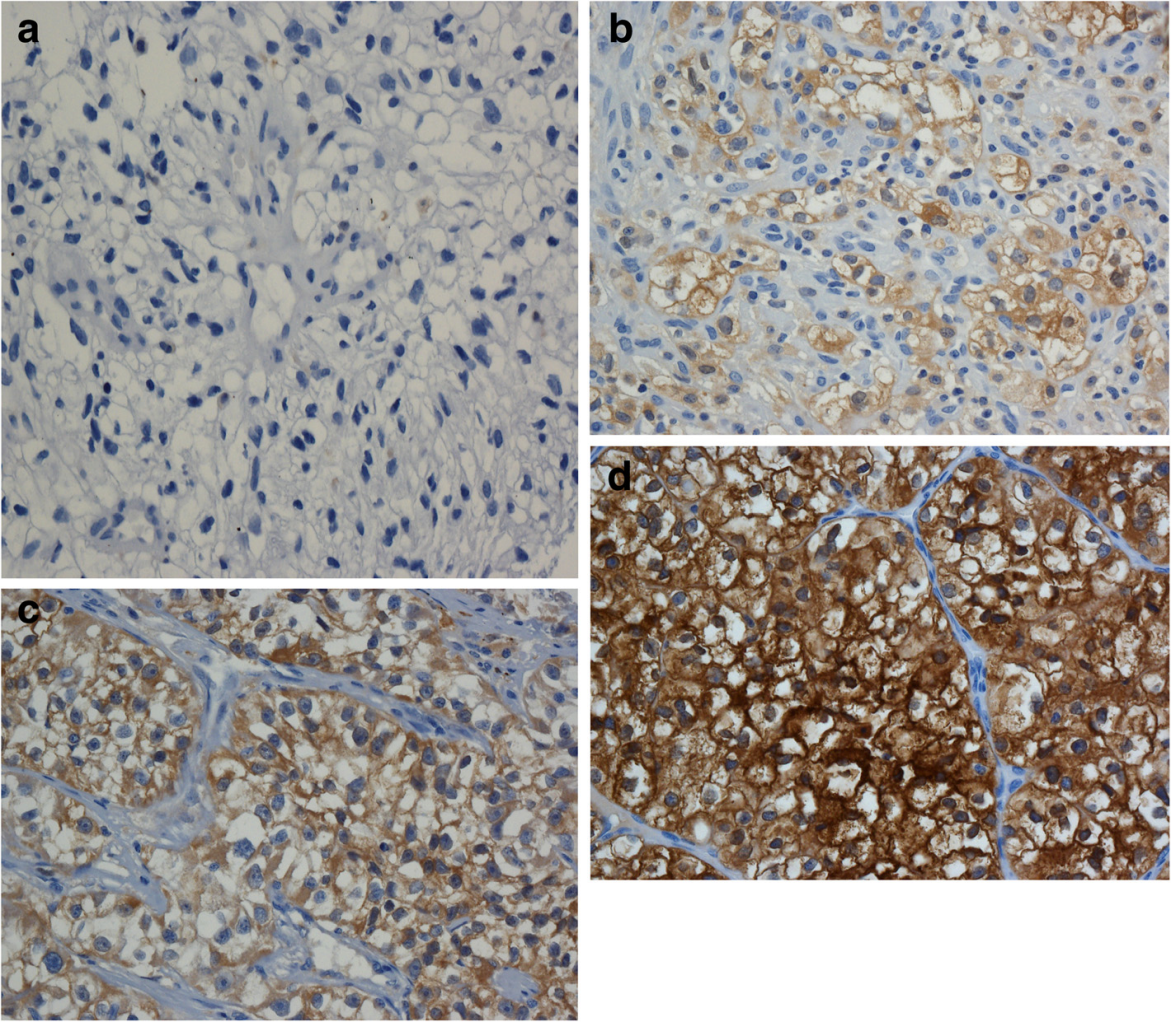
Representative example of staining intensity (a) Staining intensity 0 (no staining); (b) Staining intensity 1 (weak); (c) Staining intensity 2 (moderate); (d) Staining intensity 3 (strong).

We purchased mouse anti-human aldehyde dehydrogenase (ALDH1) monoclonal antibody from BD biosciences (Franklin Lakes, NJ, USA). Representative sections were cut from each TMA. Immunohistochemistry was performed using the Ventana automated system and VentanaIview DAB detection kit (Ventana Medical Systems Inc., Tucson, AZ, USA) at the immunohistochemistry laboratory of McGill University Health Center (Montreal, QC, Canada). Optimal dilution was determined by using serial dilutions. The stains were read by one resident (SA), and reviewed by one genitourinary pathologist (FB). Each core was graded according to the intensity of antibody staining (0 = no staining, 1 = minimal staining, 2 = moderate staining, 3 = intense staining). Figure 1 shows examples of each of the staining intensities. We multiplied the proportion of cells that stained in the core with their corresponding staining intensity, then summated for the total H score (maximum 300). If two cores were available from the same specimen then the average H score was used. We reported membrane and cytoplasmic staining separately. Analysis was carried out by a biostatistician using the SAS 9.2 software package (SAS Institute Inc., Cary, NC, USA). For all analyses, a *P* value less than 0.05 was considered to be statistically significant. Unless otherwise indicated, the Wilcoxon two sample test was employed.

## Results

### ALDH1 expression according to stage

In the first TMA, which contained 82 primary RCC specimens, average age and Fuhrman nuclear grade were both slightly higher in the high stage cohort (60.9 years versus 59.5 years and 2.5/4 versus 2/4). Twenty-nine patients were female (35%). Clinical follow-up data was available for 74 out of 82 patients. Average length of follow-up for all patients was 83 months. There was no disease-related mortality in the low stage group. There was no significant difference in H score when comparing low stage with high stage tumors, neither in terms of cytoplasmic (*P* = 0.6274) nor membranous staining (*P* = 0.2298). The clinico-pathological associations are listed in Tables 2 and 3.

**Table 2:** Clinico-pathologic features and associated cytoplasmic ALDH H scores

	**H score +/− SD**	***P*** **value**
Low stage	105.4 +/− 71.0	0.63
High stage	105.0 +/− 90.6	
Low grade	108.2 +/− 78.2	0.17
High grade	131.0 +/− 83.2	
Metastasis location		0.45
*Lymph node*	94.2 +/− 95.8	
*Brain*	89.4 +/− 93.0	
*Bone*	111.8 +/− 91.8	
*Lung*	61.9 +/− 82.6	
*Adrenal*	94.1 +/− 53.2	
Clear cell	95.5 +/− 75.8	< 0.0001
Non clear cell	194.4 +/− 102.9	
Matched		
*Primary*	120.3 +/− 57.7	0.022
*Metastasis*	86.8 +/− 58.9	
All primaries	109.7 +/− 75.8	0.041
All metastases	92.0 +/− 86.0	

SD: standard deviation.

**Table 3:** Clinico-pathologic features and associated membranous staining ALDH H scores

	**H score +/− SD**	***P*** **value**
Low stage	116.2 +/− 73.1	0.23
High stage	90.8 +/− 81.9	
Low grade	113.3 +/− 75.0	0.31
High grade	97.4 +/− 76.6	
Metastasis location		0.71
*Lymph node*	82.3 +/− 75.1	
*Brain*	85.3 +/− 110.2	
*Bone*	90.8 +/− 103.1	
*Lung*	98.0 +/− 95.9	
*Adrenal*	121.0 +/− 72.8	
Clear cell	111.0 +/− 84.2	< 0.0001
Non clear cell	24.3 +/− 39.2	
Matched		
*Primary*	119.6 +/− 70.1	0.03
*Metastasis*	78.6 +/− 72.9	
All Primaries	107.0 +/− 74.5	0.10
All Metastases	92.7 +/− 92.8	

SD: standard deviation.

### ALDH1 expression according to grade

Using the student’s *t-*test we examined the effect of tumor grade on ALDH expression in our cohort of primary tumors (first TMA). Both membrane staining and cytoplasmic staining of ALDH1 were not significantly different when low grade (Fuhrman grades 1 and 2) and high grade (Fuhrman grades 3 and 4) specimens were compared (*P* = 0.3113 for membrane staining and *P* = 0.1666 for cytoplasmic staining).

### Primary versus metastatic disease

Using all the TMAs, we examined the expression differences in the primary and metastatic setting. We looked at all patients with metastatic disease versus all primary tumors. We also looked at a matched cohort of patients who had samples from both their primary tumors and metastases (second TMA).

With regards to membrane staining, the metastatic specimens were not significantly different in their expression of ALDH1 than the primary specimens, although there was a trend towards significance (average metastasis H score = 92.7, average primary H score = 107.0 (*P* = 0.0992)). In the matched cohort, using the repeated measures analysis of variance procedure, metastatic specimens had significantly less ALDH1 staining as compared to their matched primary tumors (average metastasis H score = 78.6 and average primary H score = 119.6 (*P* = 0.0278)).

With regards to cytoplasmic staining, a similar pattern was observed. When considering all primary specimens and all metastases, metastases showed significantly less staining than the primary specimens (average metastasis H score = 92.0 and average primary H score = 109.7 (*P* = 0.0406)). Along the same lines, in the matched cohort the metastatic specimens were stained less than their primary counterparts (average primary H score = 120.3 and average primary metastasis score = 86.8 (*P* = 0.0216)).

### Analysis of clear cell subtype

When comparing the expression of ALDH1 in clear cell versus non-clear cell RCC, clear cell tumors showed significantly more membranous ALDH1 expression in comparison to non-clear cell subtypes (papillary and chromophobe) (*P* < 0.0001), but significantly less cytoplasmic expression. Because clear cell RCC has a biological origin distinct from other RCC subtypes we performed a subanalysis of outcomes, looking only at clear cell specimens (Table 4). With regards to grade, there was no significant difference in H-score with either membranous or cytoplasmic staining (*P* = 0.14 and *P* = 0.06, respectively). With regards to metastases, we saw a similar pattern with metastatic specimens having significantly less expression than primaries. An important difference emerged when comparing low stage and high stage specimens. Whereas in the entire group of RCC specimens there was no significant expression difference based on stage, in the clear cell cohort we found that low stage tumors expressed significantly more membranous ALDH1 than high stage tumors (*P* = 0.02).

**Table 4:** Clinico-pathologic features and associated cytoplasmic and membranous staining ALDH H scores - clear cell subtype

	**H score +/− SD**	***P*** **value**
Cytoplasm staining		
Low stage	96.0 +/− 60.2	0.75
High stage	90.6 +/− 80.8	
Low grade	90.8 +/− 57.7	0.06
High grade	119.0 +/− 79.5	
Matched		
*Primary*	115.3 +/− 57.3	0.10
*Metastasis*	85.6 +/− 60.2	
All Primaries	100.2 +/− 68.7	0.30
All Metastases	89.1 +/− 82.9	
Membrane staining		
Low stage	137.4 +/− 61.2	0.02
High stage	96.9 +/− 82.5	
Low grade	131.8 +/− 66.3	0.14
High grade	108.5 +/− 75.5	
Matched		
*Primary*	125.6 +/− 66.1	0.05
*Metastasis*	79.5 +/− 74.6	
All primaries	119.6 +/− 70.1	0.04
All metastases	95.0 +/− 93.4	

SD: standard deviation.

### ALDH1 expression and clinical outcomes

**Figure 2:**
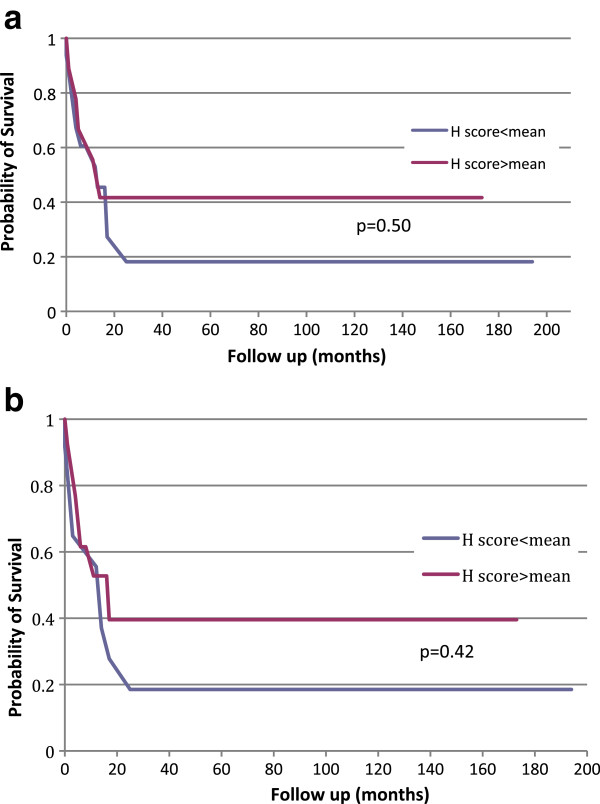
**Kaplan-Meier progression-free survival analysis of patients with primary tumors (a) Cytoplasmic staining.** Red curve is H score above mean, blue curve is H score below mean; **(b)** Membrane staining. Red curve is H score above mean, blue curve is H score below mean.

We were able to obtain clinical follow-up data for 74 out of the 82 patients in the first TMA. We decided to only examine patients with high risk (high stage) disease, because patients with T1 disease are almost always cured following nephrectomy. Indeed in our cohort all 51 patients with stage T1a disease had no evidence of recurrence at follow-up. Out of 27 patients with high risk disease (pT3/4), at the time of latest follow-up, 13 patients had died at an average of 14 months after surgery (range 6 to 29 months). Four patients remained alive but with disease recurrence and ten were alive with no evidence of disease at latest follow-up. Patients with no evidence of disease and follow-up less than one year were excluded from the analysis. When we compared patients with no evidence of disease at follow-up versus those who were alive with RCC recurrence and those who died of RCC, there was no significant difference in ALDH1 membranous or cytoplasmic expression (*P* = 0.7539 and *P* = 0.4992, respectively). We then constructed a Kaplan-Meier curve by taking the H scores of all high risk patients with clinical follow-up data, and stratified them into two groups based on the mean H score. The results of the analysis are shown in Figure 2a and b. There was no significant difference in the progression-free survival curves in terms of either cytoplasmic or membranous staining between the two groups (*P* = 0.4996 for cytoplasmic staining and *P* = 0.424 for membranous staining). When we considered only clear cell tumors, the results are unchanged with no significant expression differences between the two groups.

## Discussion

With recent advances in knowledge in cellular biology, cancer stem cells (CSCs), which form only a minority of tumoral cells, have been proven to be responsible for initiating tumors through their self-renewal capacity, generating multipotent progenitor cells. The result is an aberrant neoplastic proliferation of a population of more differentiated cancer cells which make up the bulk of the tumor [3]. It has been recently shown that the phenotype of CSCs is a reflection of both its cell of origin as well as the oncogenic transforming events [13,14]. Therefore, one approach for finding shared CSC markers was to focus on conserved stem and progenitor cell functions. In that regard, aldehyde dehydrogenase 1 (ALDH1), an enzyme responsible for the oxidation of intracellular aldehydes has been recently introduced as a potentially reliable CSC marker.

The ALDH enzymes are a group of proteins that share highly conserved sequences essential for function. Each subunit contains a catalytic domain, a cofactor binding domain, and a bridging domain. Each subtype’s catalytic pocket has a specificity for a particular substrate [15]. The ALDH1 family consists of six subtypes, three of which (ALDH1A1, ALDH1A2 and ALDH1A3) catalyze the oxidation of retinaldehyde to retinoic acid, an important regulator of gene expression [15]. Several studies have demonstrated over-expression of ALDH1 in murine and human hematopoietic and neural stem and progenitor cells, as well as the stem cell populations in multiple myeloma and acute myeloid leukemia [16-20]. ALDH activity may thus provide a common marker for both normal and malignant stem and progenitor cells. Since the expression of CSC-related markers has been shown to portend increased aggressiveness in some tumors such as pancreatic adenocarcinoma [21] and non small cell lung cancer [22] and since ALDH1 seems to be a reliable marker for CSCs, we evaluated whether ALDH1 expression could be associated with local pathologic tumor characteristics and clinical outcome in renal cell carcinoma.

To summarize our results, ALDH1 expression was not found to correlate with primary tumor grade or stage or with the clinical outcome. We did find, however, that metastatic specimens expressed significantly lower amounts of ALDH1 than primary specimens, with this result being consistent whether considering membranous or cytoplasmic staining. In our subanalysis of clear cell specimens, ALDH1 membrane expression was significantly higher in low stage as compared to high stage tumors.

Due to the paucity of studies evaluating ALDH1 expression in RCC, we compared our results to the previously reported ALDH1 expression in malignant tumors other than RCC. Table 5 summarizes the recent publications on ALDH1 expression in various malignancies. In summary, in breast cancer ALDH expression was correlated with higher tumor grade in two studies and worse clinical outcomes in one [23-25]. Similarly, an association between ALDH expression and aggressive pathological features has been reported in gallbladder adenocarcinoma in one study [26]. The findings in ovarian cancer have been contradictory, with some studies showing ALDH expression associated with poorer disease free and overall survival in serous carcinoma [27] and others showing opposite results [28]. One of the findings of the current study was that metastatic tumors had significantly lower amounts of ALDH1 expression when compared to primary tumors. This result has only been previously reported in the setting of metastatic colon cancer. Hessman *et al.* used immunohistochemistry to examine ALDH1 expression in normal, early stage, and late stage colorectal cancer, including metastases [29]. They found significantly less expression of ALDH1 in metastases compared to primary tumors. One possible explanation for decreased ALDH1 expression in the metastatic setting could be that with disease progression and metastasis, tumor cells lose the expression patterns of the normal epithelium from which they originate, and normal kidney epithelium has been shown to express large amounts of ALDH1 [30]. These findings along with ours, albeit preliminary, suggest that ALDH1 may not be a suitable target for therapy in the setting of metastatic RCC.

**Table 5:** Summary of published studies reporting the expression of ALDH1 in carcinoma

**Number of patients**	**Patient population (Publication year)**	**Tissue and methods**	**Expression compared to normal tissue**	**Association with high histologic grade**	**Association with high TNM staging**	**Expression in metastasis compared to primary**	**Prognostic factor**
**Breast cancer**							
106	Triple negative mammary carcinoma (2011) [31]	FFPE, IHC	N/A	↑ for epithelial cells - for stromal cells	N/A	N/A	↓for epithelial cells
606	Invasive mammary carcinoma, DCIS (2012) [24]	TMA, IHC	N/A	↑	-	N/A	N/A
140	2012-Triple negative mammary carcinoma [25]	TMA, IHC	N/A	-	-	N/A	↑ for stromal cells
**Gastrointestinal cancer**							
190	Gastric carcinoma (2012) [32]	FFPE, IHC	N/A	↑	↑	↑	-
96	Colorectal carcinoma (2012) [29]	FFPE, IHC	↑	N/A	↓	↓	-
1420	Colorectal carcinoma (2010) [33]	TMA, IHC	↓	+	N/A	N/A	-
186	Colorectal carcinoma (2012) [34]	TMA, IHC	N/A	N/A	N/A	N/A	↓ (for cells that were positive for beta-catenin in the nucleus)
**Gynecological cancer**							
442	Ovarian carcinoma (2009) [28]	TMA, IHC	N/A	-	↓	N/A	↑
84	Ovarian carcinoma (2012) [35]	TMA, IHC	N/A	-	-	N/A	↓
28	Serous ovarian carcinoma (2010) [36]	TMA, IHC, RT-PCR, Western	↓	N/A	N/A	N/A	N/A
98	Endometrioid ovarian carcinoma (2010) [37]	IHC	N/A	-	↑	N/A	↓
**Hepatobiliary and pancreatic cancer**							
49	Hepatocellular carcinoma (2012) [38]	FFPE, IHC	N/A	↓	-	N/A	↑
100	Gallbladder adenocarcinoma (2010) [26]	FFPE, IHC	↑	↑	N/A	N/A	↓
97	Pancreatic adenocarcinoma (2011) [39]	IHC, RT-PCR, Western	N/A	-	-	N/A	↑
78	Pancreatic adenocarcinoma (2012) [40]	TMA, IHC	N/A	N/A	N/A	N/A	-

N/A: data not available.

↑: Increased expression or positive correlation.

-: No association.

↓: Decreased expression or negative correlation.

FFPE: immunohistochemistry on formalin-fixed paraffin-embedded tissue; IHC: immunohistochemistry; RT-PCR: quantitative real time-polymerase chain reaction TMA: tissue microarray; Western: Western blot.

Another possible explanation for the differential expression of ALDH1 in metastases and primaries is based on the concept of parallel development of metastasis. Traditionally, the metastatic potential of a tumor was thought to occur as a late consequence of tumor development, through the acquisition of serial changes in tumor cells leading to the epithelial mesenchymal transition [41]. During this process cells develop the ability to break their cell-cell epithelial contacts, invade the extracellular matrix and intravasate the blood stream. In contrast, is the notion of a parallel development of metastasis in which certain cell populations exist with an inherent ability to metastasize, and which are of a separate clonal origin from the primary tumor. These cells may metastasize early on, perhaps in the subclinical stages, and do so in a manner independent from growth of the primary. Bissig *et al.* lent support to this notion by showing that 30% of RCC metastases were genetically completely different from the primary tumor, suggesting a different clonal origin [42]. Further evidence comes from the work of Schmidt-Kittler and his group who showed that in breast cancer, many patients without overt metastases had evidence of malignant cells in their bone marrow, and that these cells had fewer genetic aberrations than the primary and did not show sign of telomeric crisis [43]. This implies that dissemination may not originate from advanced clones within the primary tumor. Taken together, this evidence implies that metastases may form from completely different pathways than the primary tumor, which would explain differences in protein expression, including ALDH1.

An additional finding was that ALDH1 had a much higher degree of membrane expression in clear cell tumors as compared to other RCC types. Indeed 74% of all clear cell cores showed ≥ 30 H score whereas only 25% of papillary cores scored ≥ 30. Although classically described as a cytosolic protein, previous groups have also demonstrated membranous staining of ALDH [44,45]. Whether or not ALDH1 is actually present in the cell membrane of RCC or whether this protein is condensing near the cell membrane is impossible to determine using immunohistochemistry. Regardless of the mechanism of localization to the membrane, this increased expression may be clinically useful in subclassifying RCC as either clear cell or non clear cell.

The majority of our specimens were clear cell tumors. The most common genetic alteration in clear cell RCC is deletion of chromosome 3p, with subsequent loss of the VHL tumor suppressor gene [46]. This chromosomal loss is seen in most cases of sporadic and familial clear cell RCC but is rarely seen in other subtypes, implying a distinct biological origin of the clear cell subtype [46]. We therefore decided to conduct a subanalysis looking only at clear cell tumors. The results were mostly similar with regards to the total cohort of tumors. Namely, tumor grade did not influence the amount of ALDH1 expression, and primary specimens expressed significantly more ALDH1 then metastases. With regards to clinical outcomes, again there was no difference in progression free survival based on amount of ALDH1 staining. One important difference seen was an increased amount of membranous expression of ALDH1 in low stage as compared to high stage tumors. Similar results have also been reported by Hessman *et al.* and Chang *et al.* who found decreased ALDH1 expression in locally advanced stage colorectal cancer and ovarian cancer respectively [28,29]. As with metastatic disease, Hessman hypothesized that the negative correlation seen with locally advanced stage might be due to loss of normal expression patterns of ALDH1 seen with advanced disease.

To date there has only been one study of which we are aware which examines ALDH expression and clinical outcomes in RCC. Using a rabbit polyclonal ALDH8A1 antibody, Ozbek *et al.* examined staining patterns of 95 patients who had undergone radical or partial nephrectomy for RCC [47]. They found a positive correlation between tumor grade and ALDH expression, but did not detect a correlation with tumor stage. One possible explanation as to why this result differed from our own could be the way in which the tumors were compared by grade. In our study, tumors with Fuhrman grades 1 or 2 were compared to those with grades 3 or 4, whereas in the aforementioned study tumors with Fuhrman grades 1 or 2 were only compared to Fuhrman grade 4 tumors only. Additionally, this study used a different subtype of ALDH, ALDH8A1, which in mice kidneys shows a completely different expression pattern and different predilection for substrates than ALDH1 [48]. Hence it is plausible that in humans the two subtypes of ALDH also have different expression patterns and functions, which may explain the differing results. Finally, the antibody used was polyclonal, which may be less accurate for its target than our monoclonal antibody.

The limitation of the current study is the relatively small number of cases in which clinical follow-up was available and the fact that the immunohistochemical evaluation of ALDH1 may carry a degree of cross reactivity of ALDH1 with other ALDH subtypes and additional enzymes.

## Conclusion

We have shown that in a cohort of primary and metastatic RCC specimens, ALDH1 expression is not influenced by tumor stage or grade, but does show reduced expression in the metastatic setting. The avid expression of ALDH1 in the clear cell subtype could make it useful for the pathological classification of renal tumors. However, unlike in other cancers, ALDH1 does not seem to be a prognostic marker of RCC. Further studies are needed to determine whether the decreased ALDH1 expression in metastatic setting may be of clinical significance.

## Abbreviations

ALDH: Aldehyde Dehydrogenase; RCC: Renal cell carcinoma; TMA: Tissue microarray; CSC: Cancer stem cell.

## Competing interests

The authors declare that they have no competing interests.

## Authors’ contributions

SA participated in designing the study, scored the expression of the cores, and drafted the manuscript. KS participated in the study design and data acquisition. ST participated in designing the study and drafting of the manuscript. WK and AA participated in drafting the manuscript. JM participated in designing the study and performed immunohistochemistry. FB conceived the study, scored the expression of cores and participated in drafting the manuscript. All authors read and approved the final manuscript.
